# Gel2DE - A software tool for correlation analysis of 2D gel electrophoresis data

**DOI:** 10.1186/1471-2105-14-215

**Published:** 2013-07-06

**Authors:** Ola Kristoffer Øye, Katarina M Jørgensen, Sigrun M Hjelle, André Sulen, Dag Magne Ulvang, Bjørn Tore Gjertsen

**Affiliations:** 1 Christian Michelsen Research AS, P.O. Box 60315892 Bergen, Norway; 2 Department of Clinical Science, Hematology Section, University of Bergen, Bergen, Norway; 3 Department of Internal Medicine, Hematology Section, Haukeland University Hospital, Bergen, Norway

**Keywords:** Two-dimensional gel electrophoresis, Pixel-by-pixel analysis, Spearman rank correlation, p53 activation

## Abstract

**Background:**

Two-dimensional gel electrophoresis (2DE) is a powerful technique for studying protein isoforms and their modifications. Existing commercial 2D image analysis tools rely on spot detection that limits analysis of complex protein profiles, e.g. spot appearance/disappearance or overlapping spots. Pixel-by-pixel correlation analysis, an analysis technique for identifying relations between protein patterns in gel images and external variables, can overcome such limitations in spot analysis.

**Results:**

We have implemented the first publically available pixel-by-pixel correlation analysis tool, the software Gel2DE. 2D immunoblot time course analysis of p53 protein stabilization in response to ionizing irradiation shows that pixel-by-pixel analysis can yield an overall activation biosignature for p53, despite changing spots shape, size and position.

**Conclusions:**

Pixel-by-pixel correlation of aligned 2D images permits analysis of complex protein patterns. We anticipate that the Gel2DE correlation software will be a useful tool for future bioinformatics discoveries through 2D gel electrophoresis.

## Background

Two-dimensional gel electrophoresis (2DE) can separate complete proteins based on molecular size and charge, and thereby has a unique ability to capture detailed information about protein expression, isoforms, complex formation and post-translational modifications [1,2]. Most proteins are subject to post-translational modifications, where amino acid residues may be chemically modified or conjugated with small proteins like ubiquitin, sumo or nedd8. Proteins can also be transcribed by pre-mRNA splicing, creating different protein isoforms with varying length and amino acid composition [3]. For the separation and detection of these proteins in a single assay two-dimensional gel electrophoresis has so far proven to be the superior technology [4], robust and well suited for parallelism [1]. Most commercial software for image analysis of 2D gels still relies on detection of spots with a regular shape [5,6]. Pixel-by-pixel correlation of stacked and aligned 2D gel images may provide information that is otherwise lost and can therefore be used as an alternative to commercial methods to resolve several types of analytical problems [5].

We briefly review the underlying methodology in [5] on which our software is based: In a given population of individuals we wanted to study the relation between an external variable, e.g. chemotherapy to cancer cells or occupational benzene exposure to blood cells, and the isoform distribution and/or post-translational modification of a certain protein. We collected biological samples from the population of individuals and prepared proteins from blood cells for 2D gel electrophoresis. The sample was spiked with a denatured and fluorescently pre-labelled protein standard for accurate alignment of gel images [7]. The fluorophore-labelled proteins in this standard were selected for their molecular size and charge to ensure a standard image that covered as much of the gel as possible, enabling accurate alignment of images in a stack. These standard proteins together with the protein sample of interest were electroblotted from the SDS-PAGE gel to a membrane followed by immuno labelling and visualization by digital camera capture. The chemoluminiscent (sample) and fluorescent (standard) images of the membrane are in the rest of this report referred to as the gel signal and the gel standard images, respectively. The signal image shows the proteins to be studied, while the standard image was used for image alignment. The correlation measurement was performed by calculating the Spearman rank correlation between a chosen external variable (e.g. age, sex, survival in months) and the set of pixels at each pixel coordinate (x, y). The Spearman rank correlation is a measure of how a change in the external variable corresponds to an increase or decrease in the image pixel intensity. For the method to be applicable on categorical data, the categories must be translated to numerical values. The categories must therefore have a natural ordering in order to make the mapping to numerical values meaningful.

The Gel2DE software tool presented here is to our knowledge the first open-source application implementing a pixel-by-pixel correlation approach in a user-friendly interface. Main features include easy and intuitive alignment combined with normalisation and correlation analysis.

## Implementation: The Gel2DE application

In the following sections, we describe the implementation of the method from [5] in our software, a standalone application that can be run on a standard computer running MS Windows XP/7. We refer to the Gel2DE users’ guide [8] for a more thorough explanation of the functionality.

### Input data format

The input data format of the software requires a set of 2D gel images (PNG) for the protein signal to be studied, and a corresponding set of standard gel images. For each signal image, a number of associated external variables are subject to analysis for correlation with protein expression. The filenames of the gel images and external variables are entered into an Excel sheet that is included with the application. This Excel sheet includes a macro that generates an xml file that can be read by the Gel2DE software.

### User interface

The user is presented with the data in a graphical user interface (GUI). The GUI shows a window for the signal and standard gel images, a result window, and a table containing the external variables. The user can interactively adjust brightness and contrast of the displayed image, and can define a region of interest (ROI). The user can choose to exclude certain samples from the calculation, e.g. due to bad image quality. Work in the software is performed within the context of a “project”, which contains gel images, population parameters, settings and results directories. A project is saved as an xml file and can be loaded again at a later time.

### Alignment

Alignment of the signal images is required to handle spatial offset between gel images, and is achieved by manually aligning all images to a reference image. To avoid bias from the protein expression in alignment, separate standard gel images are used in the alignment process [7]. The software allows for interactive adjustment of transparency, so that the user can smoothly fade from the image currently under alignment to a reference image to check the alignment. The user is allowed to perform interactive rotation, scaling and translation of the image that is currently being aligned. The alignment is saved with the project.

### Normalization

Even with controlled protein concentration and under controlled lightning conditions, there will still be some gel-to-gel image variability in 2D gel electrophoresis, mainly due to manual preparation and handling of membranes. A normalization of the recorded images is therefore needed. The application implements three normalization schemes: the mean normalization, the median normalization and the Z-score normalization. The mean normalization uses the mean pixel value in each image as a normalization scale for each image. The median normalization uses the median pixel value in each image as the normalization scale for each image. The Z-score normalization implements a z-score normalization of each pixel based on the mean and the standard deviation of each image. The effect of the normalization is shown in the gel image display of the application.

### Correlation analysis

**Figure 1:**
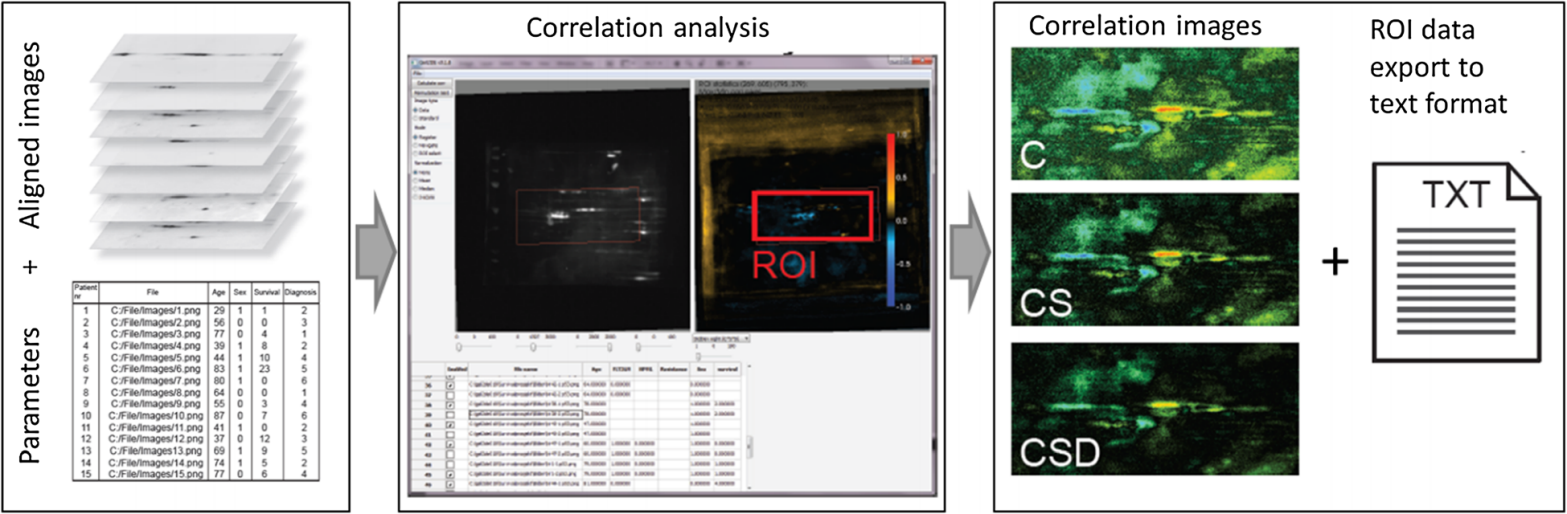
**Gel2DE work flow.** Illustration of data flow for the Gel2DE application. References to image files and corresponding external variables are read into the application from xml. In the application, the user aligns images and runs the correlation analysis. The output is correlation images and export of numerical results.

After alignment, the user selects an external variable in the GUI and runs the correlation analysis. This will result in a Spearman rank correlation value, a normalized standard deviation, and a p-value resulting from a correlation t-test or permutation test [9] for each pixel column in the gel stack. For each of these types of values an image is created. Heat map visualization is used to present the results, as shown to the right in Figure 1. In addition, the combination of calculated measures can provide information. To extract this information, we produce images where the pixel values are the product of the individually calculated values, such as correlation times standard deviation. This suppresses regions where the correlation is strong, but variations in intensity values are minor. The user can specify a ROI in the results window to investigate the analysis result quantitatively.

### Data export

The correlation values for a ROI can be exported to a text file that can be read for instance by Mat Lab [10] or R [11] for further analysis. The format of the export is given in the Gel2DE users’ guide. A set of correlation images can also be exported as a text file, including the associated settings and statistical parameters.

### Source code and software availability

The Gel2DE application is written in C++ and is tested on Microsoft Windows version XP/7. The build system is CMake, and has been tested on Microsoft Visual Studio 2008. The main frameworks used are ITK for image processing, VTK for visualization and interaction, wxWidgets for GUI and Tiny XML for xml parsing. All frameworks are cross platform compatible. A binary version of the software is available for download from [8] along with open source code (LGPL license), install instructions, a user manual and a synthetic test data set.

The software is also available for download with this article, see Additional file 1 (binary distribution) and Additional file 2 (source code).

## Results and discussion

**Figure 2:**
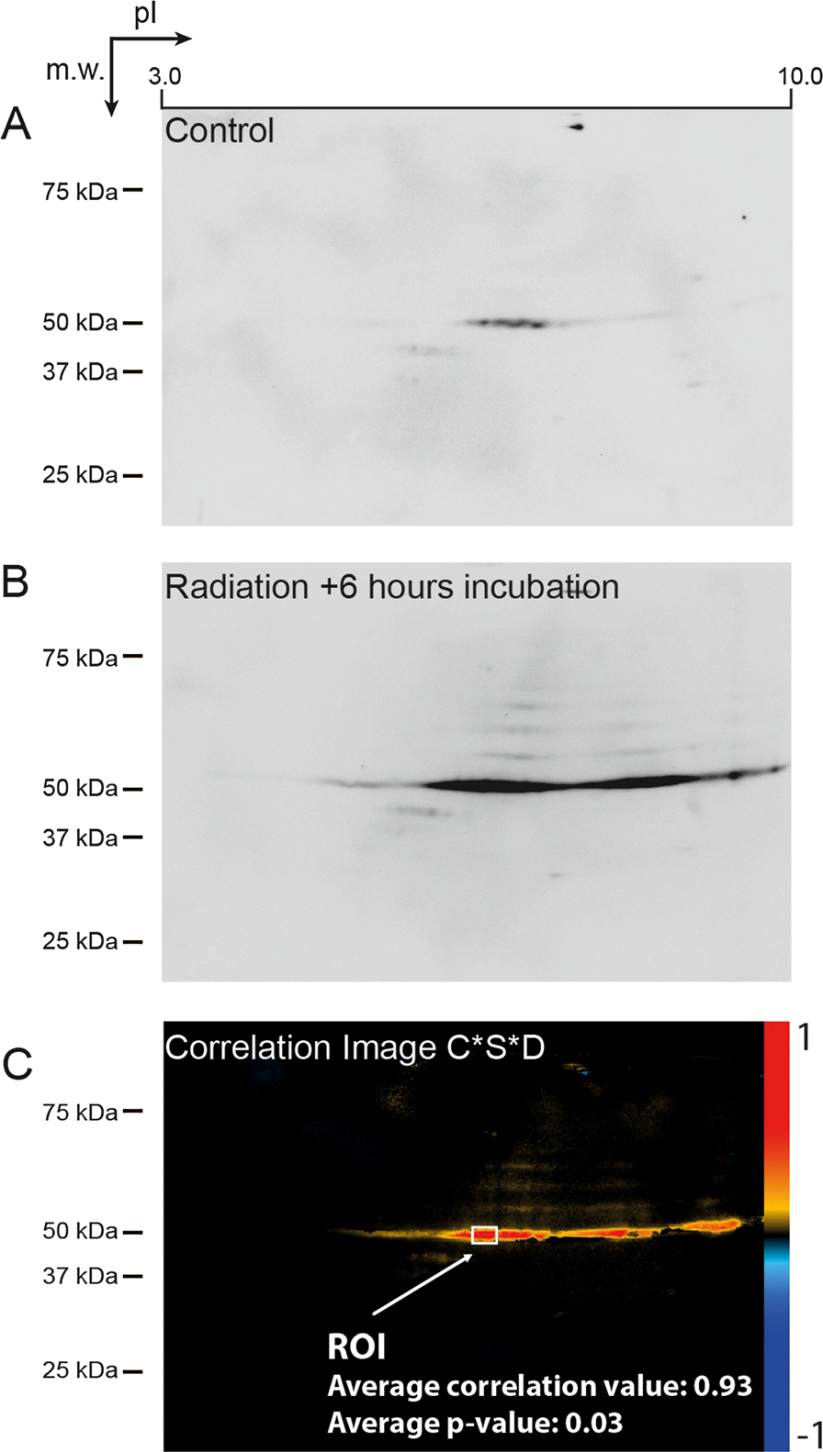
**2D gel analysis of p53 response to γ-irradiation (25 Gray, 0–8 hours). A)** Single image before treatment **B)** Single image at 6 hours post-treatment **C)** Correlation bio signature of whole p53 response time series (0, 2, 4, 6 and 8 hours). CSD = correlation coefficient * significance * variance, ROI = region of interest.

In this report we have focused on analysis of the p53 protein, a tumour suppressor protein with numerous protein modifications and where analysis by spot detection has not been feasible [5,6]. Activation of p53 using ionizing radiation is a standard way of studying p53 stabilization and subsequent activation of p53-induced genes [12,13]. An experiment demonstrating how p53 induction in a monocytic leukaemia cell line (see below) is recorded using 2D gel images was performed to demonstrate features of the analysis that make pixel-by-pixel analysis advantageous to use on such data (Figure 2).

Molm-13 cells were subjected to 25 Gray of ionizing irradiation for 8 minutes, and left to rest at 37°C, 5% CO_2_ for two, four, six and eight hours. Cells were then washed and the proteins precipitated and purified as described in [14]. Proteins were analysed by two-dimensional electrophoresis and subsequently immunoblotted with amino terminal primary antibody Bp53-12 (Santa Cruz Biotechnology) which detects p53 protein isoforms p53 full-length, p53β and p53γ [7,14,15]. Membranes were treated with luminol and stable peroxide solution (Super Signal West chemo luminescent Substrate Femto, Pierce Technology) and p53 protein expression was detected using the Kodak IS4000R.

Individual gel images, before treatment (Figure 2A) and at maximum stimulation (6 hours, Figure 2B), show typical features of p53. Before stimulation, the full-length p53 protein (at 53 kDa) is detected as a strip of five loosely interconnected spots with different sizes and shapes. These spots change their shape and size with stimulation, as well as increase in number. In fact, at 6 hours it is difficult to distinguish individual spots in the left hand tip region of p53 at all (Figure 2B). This figure also shows the characteristic streaking or laddering that probably occurs as a result of different degrees of ubiquitination in the multiple p53 molecules analysed. This is also a feature that may be removed as noise by some types of commercial software [6]. It should be noted that the “long tail” activation of full length p53 shown in Figure 2B is well developed already at two hours and remains high for the remainder of the time points (not shown). In this example, the response of the more weakly expressed p53β/γ isoforms, just visible slightly below and to the left of the full-length isoform, is overshadowed by the response in the full-length isoform.

Figure 2C demonstrates how pixel-by-pixel analysis can obtain an image representing the overall trend in the p53 response over the whole time series (0–8 hours), clearly indicating which areas of p53 are activated. In order to obtain this, the images of each time point are aligned with each other in the Gel2DE program, normalized, and then correlation analysis is performed of the gel images versus the time factor, using the workflow shown in Figure 1.

Some existing commercial software has been shown to introduce variance during image analysis [6,16]. The Gel2DE software does not use warping or harsh normalization methods. The most suitable normalization method is usually median normalization, which corrects for differences in intensity between the different images in the analysed series. As described, the software also includes a feature allowing scaling of the whole image to achieve better fits between images. Furthermore, the inclusion of all pixels in the analysis minimizes the need for warping in order to extract important information, since spots are detected even when their shapes are uneven. We have previously demonstrated that use of an improved alignment standard increases the sensitivity of feature detection, allowing the discovery of potentially novel splice variants of p53 in peripheral blood mononuclear cells in a population of more than 500 healthy volunteers [7]. Pixel-by-pixel analysis is also well suited for increased automatization of the various steps in image pre-processing as the method is further developed [6].

An additional reason why the type of activation biosignature shown in Figure 2C cannot be obtained using spot detection methods is because when p53 lengthens and shortens in response to stimulus, new spots appear and then disappear as time passes. The correlation analysis of all the images is able to find the regions that are the most strongly and consistently modified despite this. It is in fact this information – that the molecule is heavily modified towards the high PI end – that is the most important in describing the activation of p53 in response to ionizing irradiation. The average correlation value for the region of interest (ROI) of p53 with the strongest correlation is 0.93 with a statistical significance of p = 0.03. This means that the relationship between pixel intensity and time is very strong in the selected area.

Another issue that spot detection often cannot meaningfully analyse is overlapping spots [2,6]. There is no clear example of this in the experiment on p53, but this is a common problem in 2D gels – different proteins that are incompletely separated from each other. Spot detection may identify this either as one spot or as no spot at all due to a changing shape. When all the image information is retained in the analysis, it becomes possible to track the changes in both proteins despite overlapping spots [6].

The use of the software for correlation analysis of gels has also been demonstrated on 68 patients with acute myeloid leukaemia, where changes in the p53 protein biosignature were shown to correlate with survival and Flt3 receptor mutation status [15]. The correlation images obtained in this study clearly show that the method provides biosignature images indicating different strengths of correlation in different sub-regions of p53. This paper also demonstrates the possible clinical utility of the results obtained with the Gel2DE technique, as p53 is often de-regulated at the protein level in patients with acute myeloid leukaemia, and this method can indicate their responsiveness to chemotherapy and hence their treatment options and prognosis [15,17].

## Conclusion

Gel2DE is an application for performing pixel-by-pixel correlation analysis of gel electrophoresis images, and the software code has been made available to the community. The tool employs careful background correction, alignment and normalisation strategies in order to minimize the introduction of technical artefacts in results due to the data analysis itself. By preserving as much information as possible about the gel images, pixel-by-pixel analysis recovers protein features that would otherwise be lost such as chains of spots, changing spot shapes and overlapping spots. Furthermore, missing spots in images are not problematic for the attainment of a meaningful overall protein activation profile. We have employed this method to suggest new protein variants of p53 in healthy individuals and prognostication through p53 protein profiles in acute leukaemia [7,15]. We anticipate that the Gel2DE software could spur future discoveries of protein biomarkers and functionality through profiling of posttranslational modifications and isoform expression.

## Availability and requirements

**Project name: **Gel2DE

**Project home page:**http://code.google.com/p/gel2de

**Operating system(s):** Compiled for Windows 7, but uses only cross-platform frameworks, so compilation on other platforms could be considered.

**Programming language:** C++

**Other requirements:** None

**License:** LGPL

**Any restrictions to use by non-academics:** None

## Competing interests

The authors declare that they have no competing interests.

## Author's contributions

OKØ and DMU designed and developed the software, KMJ, SMH and AS have provided raw data, contributed with software specification and have been expert test users throughout the development phase. BTG initiated and led the project. All authors have read and approved the final manuscript.

## Supplementary Material

Additional file 1 **Gel2DE software distribution.** Precompiled Gel2DE executable for Windows 7, with documentation and a synthetic test data set. The distribution is also downloadable from http://code.google.com/p/gel2de. Click here for file

Additional file 2 **The Gel2DE source code.** The latest version of this source code will be available through SVN from http://code.google.com/p/gel2de. Click here for file
