# Supplementary material for: Gel2DE - A software tool for correlation analysis of 2D gel electrophoresis data
Source: BMC Bioinformatics. 2013 Jul 6;14:215. doi: 10.1186/1471-2105-14-215 (PMC3710208; doi:10.1186/1471-2105-14-215)
Supplement: Additional file 1 — Gel2DE software distribution. Precompiled Gel2DE executable for Windows 7, with documentation and a synthetic test data set. The distribution is also downloadable from http://code.google.com/p/gel2de. [file 1471-2105-14-215-S1.zip › gel2de-1.0-win7/Users manual Gel2DE v1.0.pdf]

Users manual Gel2DE v.1.0

About Gel2DE

Gel2DE is an application developed for performing correlation studies on sets of gels from two dimensional gel electrophoresis.

Installation

Create the directory where you want to install Gel2DE and unzip the downloaded distribution into the directory. Run GelCorrelation.exe to start the software. A log file (gel2de.log) with information from the running of the program will be saved in the run directory. This can be useful in case of problems with the software.

Configuration

This section describes how to prepare a project to be opened in gel2de.

Project structure

The work with gel images in Gel2DE is done in the scope of a *project*. A project is opened by opening a project file that leads to loading of gel images, clinical parameters and other project settings.

The project file

The project file defines a project, i.e. which gel images and clinical parameters that are available for analysis, how these are aligned, which gels are active, and what color table to use.

The project file primarily holds references to other files such as image files etc. A project file has to be created manually for a new project (use for instance notepad, textpad or similar). The format of the file is as follows (see examples\project.xml in the distribution):

```
<project>
    <ParameterFile>project\AML patient overview.xls.xml</ParameterFile>
    <RegistrationFile>project\registration.xml</RegistrationFile>
    <OutputDirectory>project\output</OutputDirectory>
    <ColorTransferFunction>project\ctf.xml</ColorTransferFunction>
</project>
```

The file references are specified relative to the location of the project file. The four lines must always be included in the project file:

- ParameterFile: The file that points to gel images and clinical parameters
- RegistrationFile: The file that holds the alignment data for gel images (the file need not exist, it is created if not found)
- OutputDirectory: the directory to put output from analysis

- ColorTransferFunction: defines a color table for visualization of the results

ParameterFile

Gel2DE requires a parameter xmlfile that points to a set of gel images and corresponding clinical parameters. These can be generated from an excel spreadsheet export, see exampledata\geldata.xls for an example. The spreadsheet contains a macro that exports the content and file references to an xml file that can be read by Gel2DE. It is important that the clinical parameters in the spreadsheet does not contain spaces. Special characters can also cause problems, so it is recommended to use a-z and 0-9 in parameter names. Gel2DE will only use the parameters that have numerical values, so in cases where a numeric value does not exist, these has to be translated to numbers (e.g. +/- = 1/-1).

When the spreadsheet is complete, press «Export to XML for 2DE analysis». This will generate a file (geldata.xls.xml in the example case), this needs to be referenced in the ParameterFile tag in the project file.

RegistrationFile

The registration file contains the alignment data of individual gel images. When starting a new project, this file does not need to exist, it will be created when the user selects “Save Registration” in the application. It is still important that a reference to the file is included in the project file. If the same set of gels are used in several projects, the same registration file can be used for all projects.

Output directory

The results from the correlation analysis will be put in this directory. The directory must exist when a project is loaded. The analysis will have names of the type

```
corr-<date>-<time>-<normalizationtype>-<blur level>-<parametername>
```

for instance corr-102108-091017-CD13. Naming of all files will be done this way.

ColorTransferFunction

This files specifies a user defined colortable, and is on the format:

```
<CTF>
       <CTFName>test</CTFName>
       <RGBPoint x="1" r="1" g="0" b="0"/>
       <RGBPoint x="0" r="0" g="0" b="0"/>
       <RGBPoint x="-1" r="0" g="0" b="1"/>
</CTF>
```

In this case the value 1 (x = 1) will have the color red (rgb = 1, 0, 0), the value 0 (x = 0) will be black (rgb = 0, 0, 0), while the value -1 (x=-1) will have the color blue (rgb = 0, 0, 1). The colors between the points will be interpolated. Several points can be defined on the axis if necessary.

Using the application

To open a project, select File->Open project and choose the project xml file. A list of gel images and corresponding clinical parameters will then show up, as shown in the image below. To look at a gel image, double click in the list.

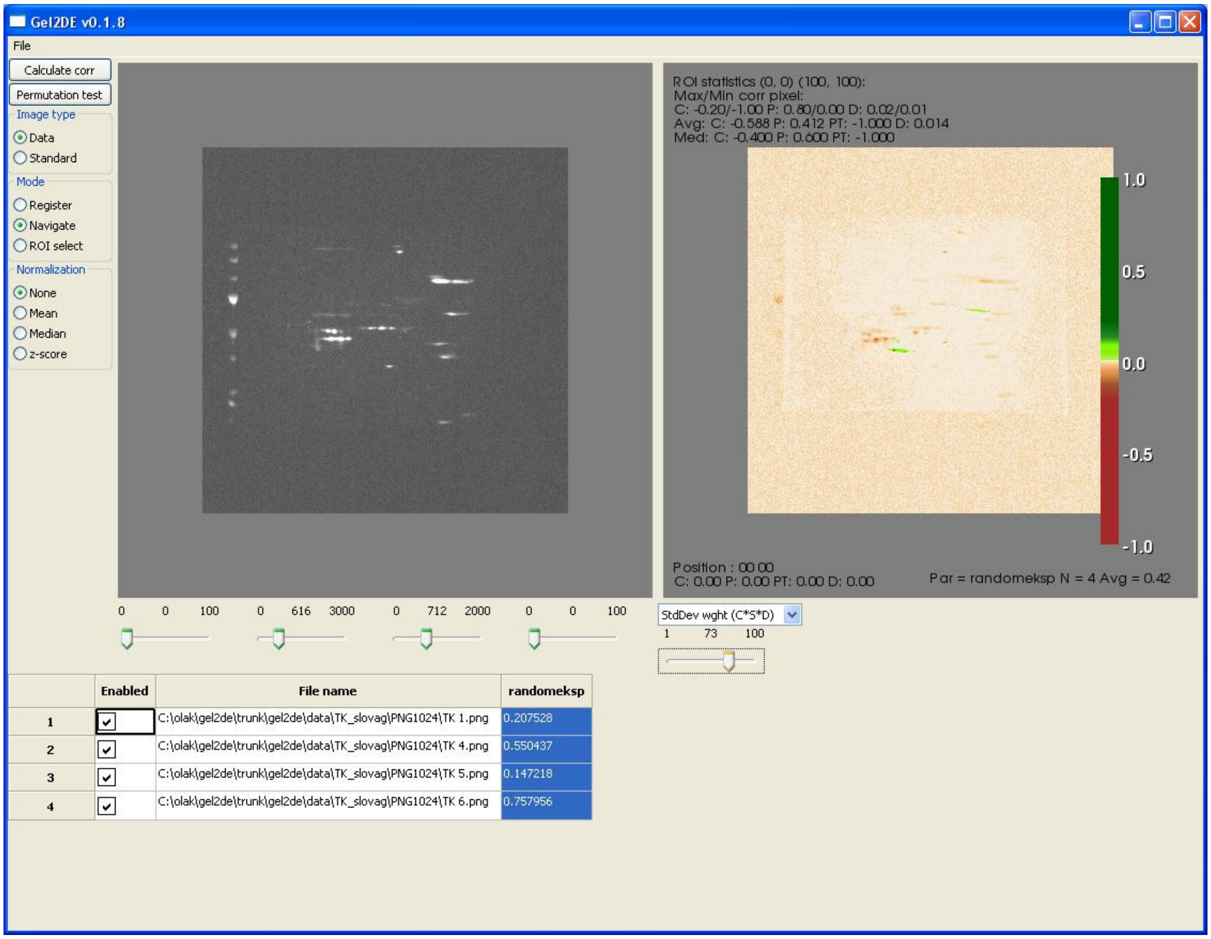

Registration, navigation and choice of ROI (Region Of Interest)

To register (align images), choose “Register” in the “Mode” box. The chosen gel image can then be moved and scaled relative to the other gels. The controls are:

- Shift + left mouse button: Move
- Ctrl + left mouse button: Rotate
- Right mouse button: Scale

To navigate in the image, i.e. move the whole “gel image stack”, choose “Navigate”. The controls work the same way as in register mode, but is applied to all gel images.

If the user wants to look at the corresponding standard image, choose «Standard» under image type. Gel2DE requires that the standard image that belongs to A.png is named A st.png.

The first gel image is always the reference image that the other gels are registered/aligned to. Pull the slider to the left under the gel image to fade over to the standard gel. This is useful to check the alignment of gels.

To choose a Region of Interest (ROI), for instance for exporting data, choose ROI mode. A ROI can then be selected in the image. The ROI can also be defined directly in the correlation image independent of the mode used. The chosen ROI is always linked between the gel image and the result display.

Adjustment of contrast and brightness in gel images

To adjust the contrast and brightness, use the two middle sliders under the gel window. These controls brightness and contrast. The effect should be immediately visible in the gel image view.

Normalization

Three image intensity normalization variants exist:

- None: No normalization, the actual pixel values are shown in the gel image and used in the correlation computation.

- Mean: For each gel, the mean pixel intensity is calculated. All the pixel values in an image are then divided by the mean pixel intensity for that image.

- Median: For each gel, the median pixel intensity is calculated. All The pixel values in an image are then divided by the median pixel intensity for that image.

- z-score: For each gel, the mean intensity and intensity standard deviation is computed. Each pixel is then calculated as follows

    Pixel intensity = (pixel intensity – mean)/stddev

The chosen method of normalization is used both in images and computations.

Smoothing

By adjusting the slider on the right side of the gel window, the amount of smoothing in the data can be adjusted. The smoothing is performed as a convolution with a Gaussian kernel. The radius of the kernel is adjusted with the slider in units of 1/10 pixels.

Correlation analaysis

When the alignment is correct, the analysis can be run. To do this, select a clinical parameter by clicking the column name. The column turns blue. Click the button “Calculate corr”. When the computation is completed, the result is shown as an image in the right window. In the combo box below the image, several display modes can be selected. The output will also be stored in the output directory specified in the project file.

Only the gels that have a value for the selected clinical parameter are included in the analysis. Gels without a value for the current clinical parameter are excluded from the correlation analysis.

A gel can also manually be excluded from analysis (for instance because of bad data) by disabling Enabled in the table. The gel will be excluded for all analysis. This is stored with the project file (File->Save project).

In the results window, correlation values for ROI and mouse pointer is shown. A ROI can be selected either directly with mouse, or by clicking File->Specify ROI. The coordinates is specified as:

<startx>,<starty>;<stopx>,<stopy>

For instance

100,120;200,300

Chosen ROI coordinates is shown on the top line in the image.

Max/Min corr pixel: The pixel with the max and min correlation value from ROI is shown as C. P value and normalized standard deviation is shown as P and D respectively.

Avg: For the selecetd ROI the display shows average correlation C, two sided p-value from t test (P), two sided p-value from permuation test (PT), and normalized standard deviation (D).

In the lower left part of the window, the current mouse coordinates and the calculated values for this pixel is shown. To the lower right, the value of the chosen parameter, the number of gels and average parameter value is shown.

Permutation test

To perform a permuation test, choose a ROI and click “Permuation Test”. The chosen parameter will then be permuted 5000 times for each pixel (NB: this might take a long time depending on the amount of data loaded). When the calculation is finished, the permutation image will be updated. The permutation test also writes a file for each pixel column, where pixel coordinates is defined in the file name. The name of the file is on the format

```
Stack column: (pixel and chosen parameter for each pixel stack)
(Normalized) intensity; external parameter
…
Measured corr: (correlation measured in original stack)
0.45
Permuted sets: (correlation values for each permutasjon)
0.32;0.12;0.04;0.14;…
Permutation p-value: (calculated p value)
0.7418
```

The final value is a two-tailed p value based on the distribution of correlations for the permuted datasets with respect to the original value.

P = (count(abs(permuted correlation) > abs(original correlation))/(total count)

Export of data

By selecting a ROI in the gel window, it is possible to export data by selecting File->Export ROI, for instance for analysis in R, Matlab or similar. Data is exported in a text format, following this format:

```
<Gel image filename 1>
540;530;… (; separated list of all normalized pixel intensities for the gel image, using current normalization scheme)
<Clinical parameter 1>;<value>
<Clinical parameter 2>;<value>
….
<empty line>
<Gel image filename 2>
….
```

It is also possible to do a screen dump of the results window by selecting “File->Result screen dump”. The result will be exported with zoom and ROI to the results directory. The filename is fixed, so previous screen dumps will be overwritten.
