# Supplementary figures and images for: Gel2DE - A software tool for correlation analysis of 2D gel electrophoresis data
Source: BMC Bioinformatics. 2013 Jul 6;14:215. doi: 10.1186/1471-2105-14-215 (PMC3710208; doi:10.1186/1471-2105-14-215)

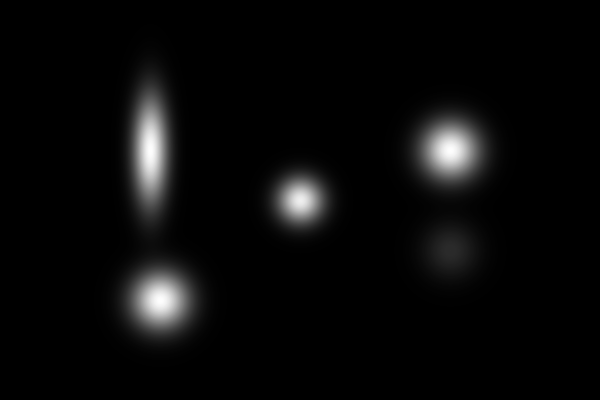

Supplement: Additional file 1 — Gel2DE software distribution. Precompiled Gel2DE executable for Windows 7, with documentation and a synthetic test data set. The distribution is also downloadable from http://code.google.com/p/gel2de. [file 1471-2105-14-215-S1.zip › gel2de-1.0-win7/exampledata/synthetic/16bit/image01 st.png]

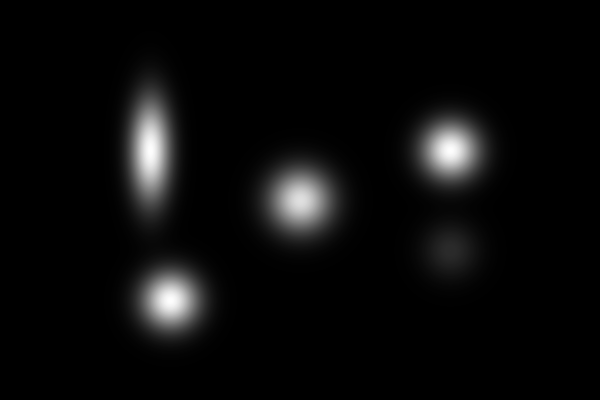

Supplement: Additional file 1 — Gel2DE software distribution. Precompiled Gel2DE executable for Windows 7, with documentation and a synthetic test data set. The distribution is also downloadable from http://code.google.com/p/gel2de. [file 1471-2105-14-215-S1.zip › gel2de-1.0-win7/exampledata/synthetic/16bit/image02 st.png]

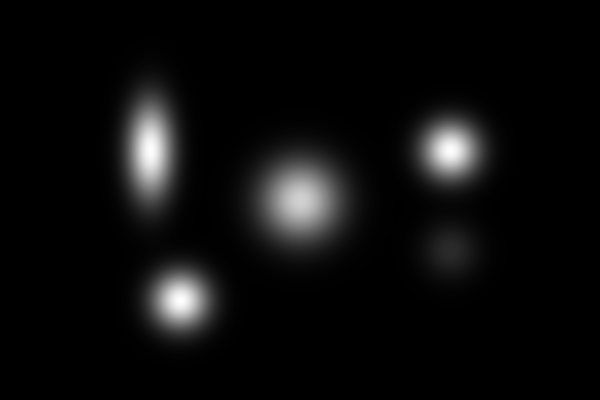

Supplement: Additional file 1 — Gel2DE software distribution. Precompiled Gel2DE executable for Windows 7, with documentation and a synthetic test data set. The distribution is also downloadable from http://code.google.com/p/gel2de. [file 1471-2105-14-215-S1.zip › gel2de-1.0-win7/exampledata/synthetic/16bit/image03 st.png]

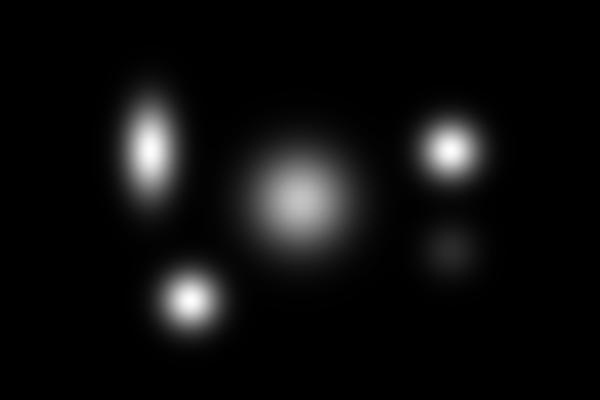

Supplement: Additional file 1 — Gel2DE software distribution. Precompiled Gel2DE executable for Windows 7, with documentation and a synthetic test data set. The distribution is also downloadable from http://code.google.com/p/gel2de. [file 1471-2105-14-215-S1.zip › gel2de-1.0-win7/exampledata/synthetic/16bit/image04 st.png]

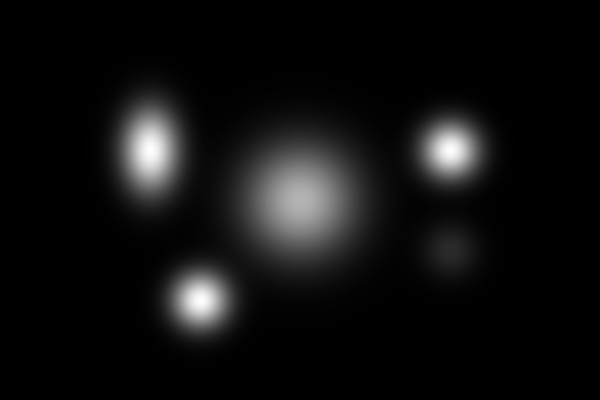

Supplement: Additional file 1 — Gel2DE software distribution. Precompiled Gel2DE executable for Windows 7, with documentation and a synthetic test data set. The distribution is also downloadable from http://code.google.com/p/gel2de. [file 1471-2105-14-215-S1.zip › gel2de-1.0-win7/exampledata/synthetic/16bit/image05 st.png]

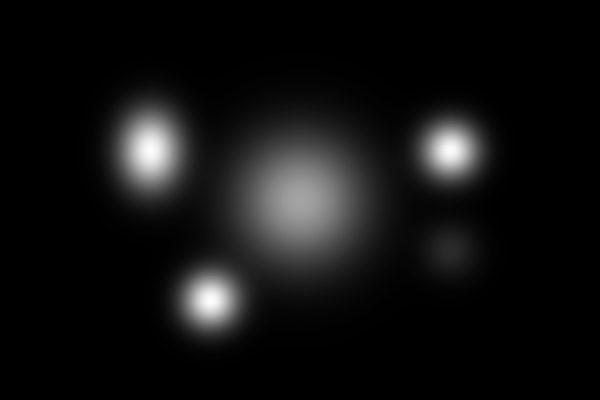

Supplement: Additional file 1 — Gel2DE software distribution. Precompiled Gel2DE executable for Windows 7, with documentation and a synthetic test data set. The distribution is also downloadable from http://code.google.com/p/gel2de. [file 1471-2105-14-215-S1.zip › gel2de-1.0-win7/exampledata/synthetic/16bit/image06 st.png]

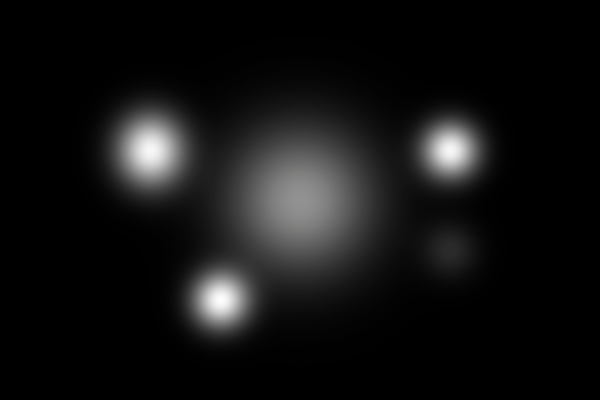

Supplement: Additional file 1 — Gel2DE software distribution. Precompiled Gel2DE executable for Windows 7, with documentation and a synthetic test data set. The distribution is also downloadable from http://code.google.com/p/gel2de. [file 1471-2105-14-215-S1.zip › gel2de-1.0-win7/exampledata/synthetic/16bit/image07 st.png]

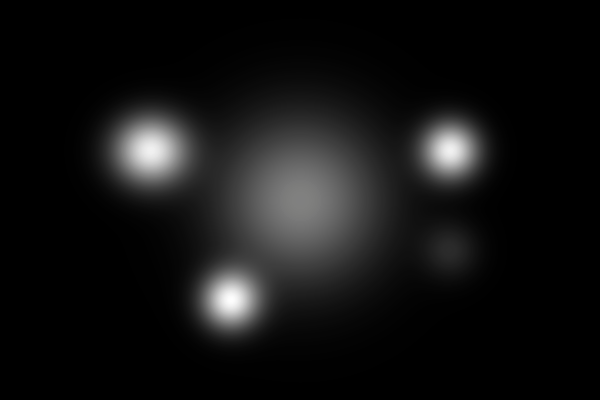

Supplement: Additional file 1 — Gel2DE software distribution. Precompiled Gel2DE executable for Windows 7, with documentation and a synthetic test data set. The distribution is also downloadable from http://code.google.com/p/gel2de. [file 1471-2105-14-215-S1.zip › gel2de-1.0-win7/exampledata/synthetic/16bit/image08 st.png]

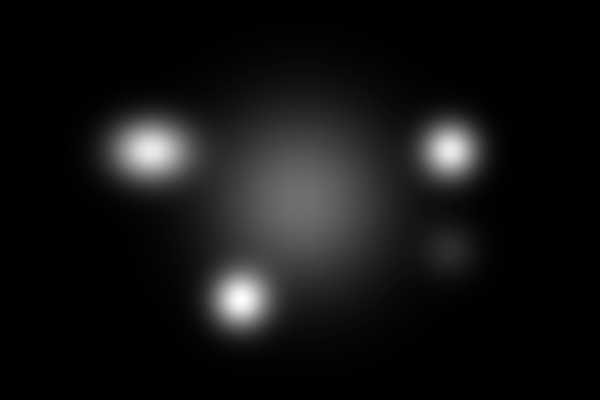

Supplement: Additional file 1 — Gel2DE software distribution. Precompiled Gel2DE executable for Windows 7, with documentation and a synthetic test data set. The distribution is also downloadable from http://code.google.com/p/gel2de. [file 1471-2105-14-215-S1.zip › gel2de-1.0-win7/exampledata/synthetic/16bit/image09 st.png]

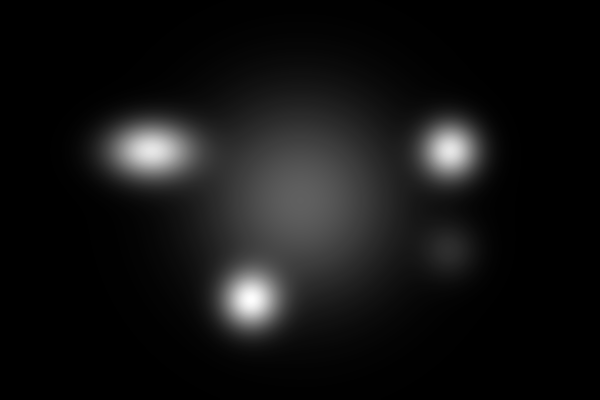

Supplement: Additional file 1 — Gel2DE software distribution. Precompiled Gel2DE executable for Windows 7, with documentation and a synthetic test data set. The distribution is also downloadable from http://code.google.com/p/gel2de. [file 1471-2105-14-215-S1.zip › gel2de-1.0-win7/exampledata/synthetic/16bit/image10 st.png]

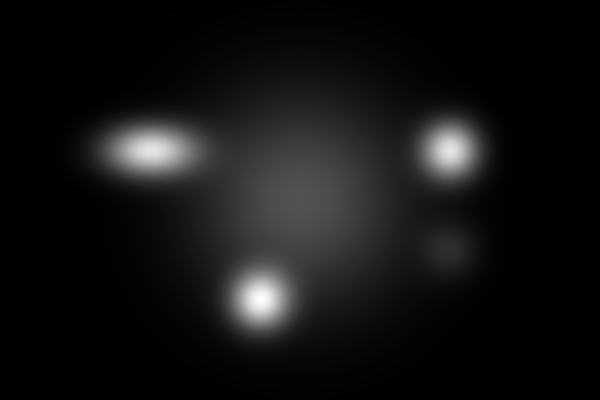

Supplement: Additional file 1 — Gel2DE software distribution. Precompiled Gel2DE executable for Windows 7, with documentation and a synthetic test data set. The distribution is also downloadable from http://code.google.com/p/gel2de. [file 1471-2105-14-215-S1.zip › gel2de-1.0-win7/exampledata/synthetic/16bit/image11 st.png]

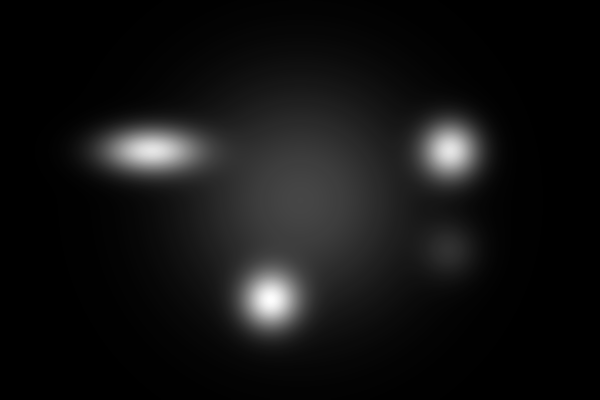

Supplement: Additional file 1 — Gel2DE software distribution. Precompiled Gel2DE executable for Windows 7, with documentation and a synthetic test data set. The distribution is also downloadable from http://code.google.com/p/gel2de. [file 1471-2105-14-215-S1.zip › gel2de-1.0-win7/exampledata/synthetic/16bit/image12 st.png]

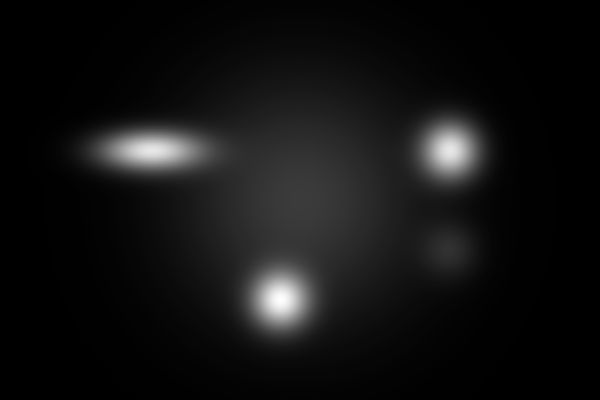

Supplement: Additional file 1 — Gel2DE software distribution. Precompiled Gel2DE executable for Windows 7, with documentation and a synthetic test data set. The distribution is also downloadable from http://code.google.com/p/gel2de. [file 1471-2105-14-215-S1.zip › gel2de-1.0-win7/exampledata/synthetic/16bit/image13 st.png]

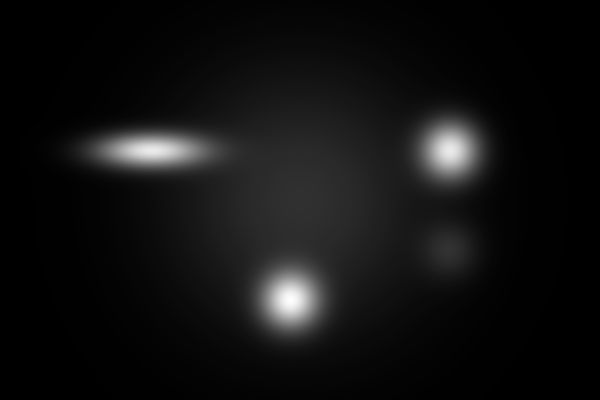

Supplement: Additional file 1 — Gel2DE software distribution. Precompiled Gel2DE executable for Windows 7, with documentation and a synthetic test data set. The distribution is also downloadable from http://code.google.com/p/gel2de. [file 1471-2105-14-215-S1.zip › gel2de-1.0-win7/exampledata/synthetic/16bit/image14 st.png]
