# Supplementary material for: Gel2DE - A software tool for correlation analysis of 2D gel electrophoresis data
Source: BMC Bioinformatics. 2013 Jul 6;14:215. doi: 10.1186/1471-2105-14-215 (PMC3710208; doi:10.1186/1471-2105-14-215)
Supplement: Additional file 2 — The Gel2DE source code. The latest version of this source code will be available through SVN from http://code.google.com/p/gel2de. [file 1471-2105-14-215-S2.zip › doc/gel2de_userguide_v.1.0.0.doc]

Bruksanvisning Gel2DE v.0.1.8

# 1 Om Gel2DE

Gel2DE er et program utviklet for å gjøre korrelasjonsstudier av geler fra to-dimensjonal gel elektroforese.

# 2 Installasjon

Opprett katalogen der Gel2DE skal installeres. Pakk opp gel2de[dato].zip i denne katalogen. For å starte, dobbeltklikk GelCorrelation.exe. En loggfil (gel2de.log) med informasjon fra kjøringen av programmet blir lagret i katalogen programmet kjøres fra. Denne kan være nyttig dersom det problemer med å starte programmet eller å laste et prosjekt.

# 3 Konfigurasjon

## 3.1 Prosjektstruktur

Arbeidet med geler i gel2DE gjøres innenfor et prosjekt. Et prosjekt åpnes ved å åpne en prosjektfil, som fører til innlasting av geler, kliniske parametere og andre settinger.

### 3.1.1 Prosjektfilen

Prosjektfilen definerer et prosjekt, dvs hvilke geler og kliniske parametere som er tilgjengelig i analysen, hvordan disse er registrert, hvilke geler som er aktive, og hvilken fargetabell som skal brukes.

Prosjektfilen inneholder hovedsakelig referanser til andre filer. En prosjektfil må opprettes manuelt for et nytt prosjekt (bruk for eksempel textpad, notpad eller lignende), og er på formatet (se i examples\project.xml i gel2de.zip)

<project>

<ParameterFile>C:\gel2de\project\AML patient overview.xls.xml</ParameterFile>

<RegistrationFile>C:\gel2de\project\registration.xml</RegistrationFile>

<OutputDirectory>C:\gel2de\project\output</OutputDirectory>

<ColorTransferFunction>C:\gel2de\project\ctf.xml</ColorTransferFunction>

</project>

Disse fire linjene må alltid være med i prosjektfilen.

- **ParameterFile**: filen med geler og kliniske parametere.
- **RegistrationFile**: inneholder plasseringen av de individuelle gelene (trenger ikke finnes når et nytt prosjekt starter).
- **OutputDirectory**: vil inneholde resultatene av analyser (må opprettes manuelt)
- **ColorTransferFunction**: definerer en fargetabell for resultater.

En god praksis kan være å plassere prosjektfilen og de andre filene i samme katalog, men dette er helt opp til brukeren.

### 3.1.2 ParameterFile

Gel2DE krever en pasientparameter-xmlfil som består av en liste av geler og tilhørende kliniske parametere. Denne kan genereres fra et regneark, se examples\AML patient overview.xls. Regnearket inneholder en makro som kan eksportere innholdet til en xml fil som leses av Gel2DE.

Det er viktig at parameternavnene i regnearket ikke inneholder mellomrom. Det kan også se ut som om norske tegn i noen tilfeller kan forårsake problemer, så unngå dette dersom det er mulig.

Gel2DE vil bare bruke parametere som har numeriske verdier, så i de tilfeller der en numerisk verdi ikke finnes, må disse oversettes av brukeren av regnearket på en meningsfull måte (for eksempel:

- = 0 og + = 1).

Når regnearket er klart, trykk ”Export to XML for 2DE-analysis”. Dette vil generere en fil (AML patient overview.xls.xml i eksempelet), som skal pekes på av ParameterFile tagen i prosjektfilen.

### 3.1.3 RegistrationFile

Denne filen inneholder registreringen av individuelle geler. Ved oppstart av et nytt prosjekt trenger ikke denne filen finnes, den vil genereres når man i applikasjonen velger Save Registration. Det er likevel viktig at en sti for denne er definert i prosjektfilen.

Dersom det samme settet av geler inngår i flere prosjekt, kan den samme registreringsfilen brukes for alle prosjektene.

### 3.1.4 Output directory

Resultater fra korrelasjonsanalysene vil havne i underkataloger av denne katalogen. Katalogen må finnes når prosjektet lastes. Studiene vil havne i kataloger med navn av typen

corr-<dato>-<tid>-<normaliseringsmetode>-<blur level>-<parameternavn>

for eksempel corr-102108-091017-CD13. Navngivning av resultatfiler vil også gjøres ut fra dette.

### 3.1.5 ColorTransferFunction

Denne filen inneholder en brukerdefinert fargetabell, og er på formatet:

<CTF>

<CTFName>test</CTFName>

<RGBPoint x="1" r="1" g="0" b="0"/>

<RGBPoint x="0" r="0" g="0" b="0"/>

<RGBPoint x="-1" r="0" g="0" b="1"/>

</CTF>

I dette tilfellet vil verdien 1 (x=1) få fargen rød (rgb = 1, 0, 0), verdien 0 (x = 0) vil bli svart (rgb = 0, 0, 0), og verdien -1 (x=-1) vil får fargen blå (rgb = 0, 0, 1). Fargene imellom vil bli interpolert. Det kan defineres flere punkter på fargeskalaen dersom ønskelig.

Dersom filen ikke er definert i prosjektfilen, vil en default fargetabell brukes.

# 4 Praktisk bruk

For å åpne et prosjekt, velg File->Open project og velg et prosjekt.

En liste med geler og tilhørende kliniske parametere vil da vises.

For å se på geler, dobbeltklikk i listen.

## 4.1 Registrering, navigering og valg av ROI (Region Of Interest)

For å registrere, velg ”Register” under mode. Den valgte gelen kan da flyttes og skaleres relativt til de andre gelene. Kontrollerne er:

- Shift + venstre musknapp: Flytt
- Ctrl + venstre musknapp: Roter
- Høyre musknapp: Skaler

For å navigere i bildet, dvs flytte selve ”gel stacken”, velg navigate. Kontrollerne fungerer på samme måte som i Register mode.

Dersom man vil se det tilhørende standardbildet, velg standard under image type. Gel2DE forventer at standardbildet som tilhører bilde A.tif heter A st.tif. Dersom dette ikke finnes, kan det forårsake problemer.

Gel1 er alltid referanse gelen som de andre gelene skal registreres inn i. Dra i slideren lengst til venstre under gel imaget for å fade over i referanse gelen. Dette er nyttig for å sammenligne registreringen av gelene.

For å velge en region of interest, for eksempel for export, velg ROI mode. ROI kan da velges i gel bildet. ROI kan også velges direkte i korrelasjonsbildet uavhengig av hvilken mode som brukes. Den valgte ROI synkroniseres alltid mellom gel og resultatvisningen.

## 4.2 Justering av kontrast og lysstyrke i geler

For å justere kontrast og lysstyrke brukes de to midterste sliderene under gel vinduet. Disse følger Window/Level, der venstre slider justerer level (lysstyrke) og høyre window (kontrast)

## 4.3 Normalisering

Tre normaliseringsvarianter finnes:

- **None**: Ingen normalisering, de faktiske pixelverdiene vises i gel vinduet og brukes i korrelasjonsberegningen
- **Mean**: For hver gel regnes gjennomsnittlig pixel intensitet ut. Alle pixlene i en gel deles så på denne verdien. Dette gjøres individuelt for hver gel.
- **Median**: For hver gel regnes median intensitet ut. Alle pixlene i en gel deles så på denne verdien. Dette gjøres individuelt for hver gel.
- **z-score:** For hver pixel regnes
  - pixel intensitet = (pixel intensitet – xmean)/stddev

Hvor xmean er gjsnitt intensitet i bildet og stddev er intensitet std dev i bildet.

NB: Den valgte normaliseringsmetoden brukes i visninger, beregninger og eksport av data.

## 4.4 Glatting

Ved å dra i slideren til høyre under gel vinduet kan man justere mengden av glatting av gel data. Glattingen utføres som konvolusjon med en gaussisk kjerne. Radiusen på kjernen justeres med slideren lengst til høyre i enhet av 1/10 pixels.

## 4.5 Korrelasjonsanalyse

Når registreringen er riktig, kan selve analysen kjøres. For å gjøre dette, velg et klinisk parameter ved å klikke på kolonnenavnet. Kolonnen blir da blå (CD13 i figur). Trykk på knappen ”Calculate corr”. Når beregningen er ferdig, vil resultatet vises i bildet til høyre. I boksen under kan man velge ulike måter å se resultatet på. Disse finnes også under output katalogen som er spesifisert i prosjektfilen.

Merk at bare numeriske verdier for kliniske parametere blir godtatt. De geler som ikke har numerisk verdi for et gitt parameter vil utelates i en korrelasjonsanalyse for parameteret.

Geler kan også utelukkes fra en analyse ved å klikke av Enabled i tabellen. Da vil gelen utelates for alle analyser. Dette lagres med prosjektet (File->Save project).

Resultatfiler fra korrelasjonsanalysen lagres på katalogen definert i prosjektfilen som output directory.

I resultatvinduet vises verdier for ROI og muspeker.

ROI kan velges enten med mus eller ved å velge File->SpecifyROI. Koordinatene for ROI må da oppgis som

<startx>,<starty>;<stopx>,<stopy>

For eksempel

100,120;200,300

Valgt ROI koordinater vises på øverste linje i bildet.

**Max/Min corr pixel:** Pixelen med max og min korrelasjonsverdi finnes fra ROI og vises som C. P value og normalisert standardavvik for disse vises som henholdsvis P og D.

**Avg:** For ROI vises gjennomsnittlig korrelasjon C, two sided p-value fra t test (P), two sided p-value fra permutasjonstest (PT), og normalisert standard avvik (D)

Nederst til venstre i vinduet vises nåværende pixelkoordinater og de beregnede verdiene for denne pixel stacken. Nederst til høyre vises valgt parameter, antall geler og gjennomsnittlig parameterverdi.

### 4.5.1 Permutasjonstest

For å utføre permutasjonstest, merk en Region Of Interest og klikk ”Permutation test”. Det valgte eksterne parameteret vil da bli permutert og regnet korrelasjon for 5000 ganger for hver pixel. Når beregning er ferdig, vil permutasjontest resultatbildet bli oppdatert. Permutasjonstesten skriver også ut en fil for hver pixelstack, der pixel koordinater for stacken finnes i filnavnet. Filen er på formatet:

Stack column: (*pixel og valgt parameter for pixel stack)*

(normalisert)intensitet; ekstern parameter

….

Measured corr: (*korrelasjon målt i original stack)*

0.45

Permuted sets: (*korrelasjonsverdier for hver permutasjon)*

0.32;0.12;0.04;0.14;…

Permutation p-value: (*beregnet p verdi)*

0.7418

Det regnes til slutt ut en two-tailed p verdi basert på distribusjonen av korrelasjon for de permuterte datasettene i forhold til den opprinnelige verdien:

P = (count(abs(perm corr) > abs(reell corr))/(total count)

## 4.6 Eksport av data

Ved å merke en ROI i gel vinduet kan man eksportere data ved å velge File->Export ROI. En dialogboks vises for valg av filnavn, og data eksportes på tekstformat. Data følger dette formatet:

<Filnavn 1>

540;530;… ( *semikolon separert liste over alle normaliserte pixel verdier (bruker valgt normalisering))*

<Eksternt parameter 1>;<verdi>

<Eksternt parameter 2>;<verdi>

….

<tom linje>

<Filnavn 2>

….

Det kan også gjøres en screen dump av resultatvinduet ved å velge ”File->Result screen dump”. Resultat vinduet vil da bli eksportert med zoom, ROI osv til den nåværende resultatkatalogen. Filnavnet er fast, så dersom man vil beholde bilder fra flere eksporter må bildene renames manuelt.
